# Comparative Study on Seed Characteristics, Antioxidant Activity, and Total Phenolic and Flavonoid Contents in Accessions of *Sorghum bicolor* (L.) Moench

**DOI:** 10.3390/molecules26133964

**Published:** 2021-06-29

**Authors:** Bimal-Kumar Ghimire, Ji-Won Seo, Chang-Yeon Yu, Seung-Hyun Kim, Ill-Min Chung

**Affiliations:** 1 Department of Crop Science, College of Sanghuh Life Science, Konkuk University, Seoul 05029, Korea; bimalg12@konkuk.ac.kr (B.-K.G.); kshkim@konkuk.ac.kr (S.-H.K.); 2 Bioherb Research Institute, Kangwon National University, Chuncheon 24341, Korea; seojiwon789@naver.com (J.-W.S.); cyyu@kangwon.ac.kr (C.-Y.Y.)

**Keywords:** *Sorghum bicolor*, seed characteristics, antioxidant properties, total phenolic contents, flavonoid contents

## Abstract

Sorghum is a major cereal food worldwide, and is considered a potential source of minerals and bioactive compounds. Its wide adaptive range may cause variations in its agronomic traits, antioxidant properties, and phytochemical content. This extensive study investigated variations in seed characteristics, antioxidant properties, and total phenolic (TPC) and flavonoid contents (TFC) of sorghum collected from different ecological regions of 15 countries. The antioxidant potential of the seed extracts of various sorghum accessions was determined using 1,1-diphenyl-2-picrylhydrazyl (DPPH) and 2,2′-azinobis 3-ethylbenzothiazoline-6-sulfonate (ABTS) radical scavenging assays. Significant variations in TPC were observed among the sorghum accessions. All 78 sorghum accessions used in this study exhibited significant variations in TFC, with the lowest and highest amount observed in accessions C465 and J542, respectively. DPPH scavenging potential of the seed extracts for all the accessions ranged from 11.91 ± 4.83 to 1343.90 ± 81.02 µg mL^−1^. The ABTS assay results were similar to those of DPPH but showed some differences in the accessions. Pearson’s correlation analysis revealed a wide variation range in the correlation between antioxidant activity and TPC, as well as TFC, among the sorghum accessions. A wide diversity range was also recorded for the seed characteristics (1000-seed weight and seed germination rate). A dendrogram generated from UPGMA clustering, based on seed traits, antioxidant activity, TPC, and TFC was highly dispersed for these accessions. Variations among the accessions may provide useful information regarding the phytoconstituents, antioxidant properties, and phytochemical contents of sorghum and aid in designing breeding programs to obtain sorghum with improved agronomic traits and bioactive properties.

## 1. Introduction

Sorghum (*Sorghum bicolor* L.) Moench, belonging to the Poaceae family and inhabiting arid and semi-arid tropical regions, is one of the most important crops worldwide. It is recognized as an excellent source of nutrition, containing minerals, starch, proteins, vitamin E bioactive compounds, phytosterols, and policosanols, in addition to having a high fiber content [1,2,3,4,5,6,7,8]. Compared to other main crops such as rice, wheat, and corn, sorghum is ranked fifth in terms of crops that are the most produced and consumed globally [9]. The average production cost of sorghum is low. Further, a high resistance to high temperature and drought stress makes it an important food and feed crop [10,11]. Owing to its wide range of adaptability and ease of growth, sorghum is largely produced in Asian and African countries, and is a good alternative source of animal feed and human food [3,12]. Moreover, it contains various nutrients and bioactive compounds [13,14], such as phenolic acids (ferulic, tannic, and *p*-coumaric acids), flavonoids (luteolin, apigenin, catechin gallate, and epigallocatechin [15,16]), tannins [3,17], and lipids [18]. The presence of these valuable bioactive compounds may be responsible for its high LDL inhibition potential [19,20], antioxidant and antibacterial effects [21], anticancer activity that prevents gastrointestinal tract cancer [22,23,24,25], and role in preventing obesity, diabetes, and cardiovascular diseases, in addition to reducing hypertension [5]. Moreover, there is an increased interest in identifying the active ingredients in plants that can act as natural antioxidants and contribute to food preservation without damaging human health and the environment [26].

The stability of sorghum production, as well as its agronomic traits, chemical composition, and antioxidant properties may vary owing to the strong influence of abiotic factors during the growing stages. Authors of previous studies attributed the variation in the antioxidant properties of plant accessions to differences in geographical origin and genetic variations [27,28]. In addition, variations in precipitation, temperature, altitude, and edaphic factors influence the mineral content and phytochemical accumulation in plants [29,30]. Changes in the quality and composition of phytochemicals and biological activity of plant accessions that have originated and adapted to a broad geographical range have been reported by Awika et al. [17], Choi et al. [31], Davila-Gomez et al. [32], Luo et al. [33], Rhodes et al. [34], and Kil et al. [21]. However, all earlier reports were based on a small number of accessions collected from single/particular locations. To the best of our knowledge, comprehensive and comparative studies on multiple accessions concerning antioxidant activity and the TPC and TFC of sorghum have not been reported. Therefore, sorghum accession characterization is an important step for breeding programs to investigate novel and superior traits, which are useful for improving the agronomic traits of crops with increased abiotic and biotic resistances [35,36].

Phytochemicals such as phenolic acids and flavonoids are the most diverse sources of natural antioxidants, commonly present in plant species, and are typically safe for consumption as dietary supplements [37,38,39]. Several studies have shown that the disease preventive potential of plant-based dietary food is associated with the polyphenol constituents present in the plant [40]. ROS overproduction during the metabolic process has been implicated in various degenerative diseases, including diabetes, heart diseases, Alzheimer’s disease, and aging [41]. Phenolic compounds play an important role in scavenging radicals by absorbing and neutralizing oxygen and peroxide radicals [42]. Importantly, phenolic acids play an important role in preventing numerous degenerative diseases [43]. Flavonoids also possess significant antioxidant properties [44,45,46,47,48]. Besides their antioxidative properties, these compounds play a significant role in plant adaptation under different growth conditions [49,50,51,52,53,54,55,56,57]. Oxidation causes degradation of food quality and nutrition levels [24]. Numerous synthetic antioxidant compounds are used in the health sector and food industries for scavenging free radicals; however, the application of these compounds has been limited, owing to their toxicity and possible deleterious health effects [58].

Based on this hypothesis, the objectives of the present study were to determine the TPC, TFC, and antioxidant activity of sorghum accessions collected from 15 different countries. An additional aim of the study was to determine the correlation between the 1000-seed weight and germination rate, and that of TPC and TFC of accessions with antioxidant properties. This would help breeders select new accessions based on their useful traits, high concentrations of bioactive compounds, and health benefits.

## 2. Results

### 2.1. Percentage of Seed Germination and 1000-Seed Weight

**Figure 1:**
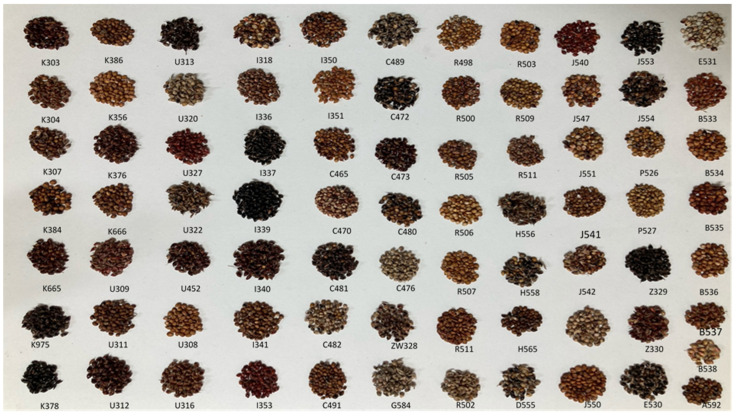
Diversity in *S. bicolor* accessions with different origins based on seed characteristics.

In this study, the mean germination percentage of *S. bicolor* accessions showed a wide variation range with geographical origin and ranged from 8 to 100% (Table 1). In comparison, the majority of accessions with higher germination rates showed different seed colors (Figure 1). Of the 78 accessions, 34 showed high germination rates of 90–100%. Of the accessions with high germination rates, three (J543, A592, and K378) achieved 100% germination whereas six, including P526, Z330, E530, I337, I341, and R506, had the lowest germination rates (less than 50%). As shown in Table 1, the germination rate of seeds was more than 90% in most accessions from Korea, the USA, China, and Russia. Conversely, a poor germination rate was observed in accessions from the Philippines. A single accession from Australia (A592, D555, and G584) had high germination rates (100%, 98%, and 94%, respectively). 

In this study, the mean germination percentage of *S. bicolor* accessions showed a wide variation range with geographical origin and ranged from 8% to 100%. In comparison, the majority of accessions with higher germination rates showed different seed colors (Figure 1). Of the 78 accessions, 34 showed high germination rates of 90–100%. Of the accessions with high germination rates, three (J543, A592, and K378) achieved 100% germination whereas six, including P526, Z330, E530, I337, I341, and R506, had the lowest germination rates (less than 50%). As shown in Table 1, the germination rate of seeds was more than 90% in most accessions from Korea, the USA, China, and Russia. Conversely, poor germination rate was observed in accessions from the Philippines. A single accession from Australia (A592, D555, and G584) had high germination rates (100%, 98%, and 94%, respectively).

### 2.2. Total Phenolic Content (TPC)

Significant variations in TPC were observed among the sorghum accessions collected from the 15 countries, ranging from 9.97 ± 0.05 to 333.58 ± 1.49 mg GAE g^−1^ of dry material (Table 2). The highest TPC was observed in the J551 accession, accounting for 333.58 ± 1.49 mg GAE g^−1^ of dry material. Variations in TPC were also observed within the accessions of different countries. For instance, a wide TPC range was observed in the Korean accessions, ranging from 18.98 ± 0.68 to 171.50 ± 3.80 mg GAE g^−1^ of dry material. A similar trend was observed in the accessions collected from America, China, Japan, India, Russia, Botswana, Hungary, Philippines, South Africa, Ethiopia, the United Kingdom, Germany, Australia, and Zimbabwe. When compared with accessions from other countries, the Chinese accessions showed the highest TPC, followed by the Russian accessions. This is especially true for accessions C80, C472 and C476, which had exceptionally high TPC (299.52 ± 2.72, 268.82 ± 0.95 and 259.52 ± 2.71 GAEg^−1^ of dry material, respectively). Compared with the TPC of accessions from different country samples, except I318, I341, and I336, the TPC of the Indian SB accessions was low; the lowest TPC was observed in the I339 accession. Single accessions collected from Australia, Germany, and Zimbabwe contained higher TPC (188.55 ± 1.35, 141.14 ± 1.22, 149.61 ± 1.63, mg GAE g^−1^ of dry material, respectively) than that from the United Kingdom (50.60. 0.41 mg GAE g^−1^ of dry material). 

### 2.3. Total Flavonoid Content (TFC)

From the ANOVA analysis, the sorghum accessions collected from different countries exhibited a significant variation in the TFC, which can be used in crop improvement programs. The flavonoid contents of the sorghum accessions are presented in Table 2. All 78 accessions of sorghum used in this study showed significant variation in TFC, ranging from 10.35 ± 0.07 to 252.67 ± 24.99 (mg QE g^−1^) of dry material, with the lowest and highest content observed in accessions C465 and J542, respectively. Variations in TFC were also observed within the accessions of different countries. For instance, a low variation in TFC content was observed in the Russian accessions (ranging from 22.97 ± 1.29 to 63.34 ± 1.46 mg QE g^−1^). Statistical analysis revealed no significant difference (*p* > 0.05) in TFC in the R498, R506, and R502 accessions. In contrast, a large variation in TFC was observed in the accessions originating from Japan (ranging from 17.33 ± 0.13 to 252.67 ± 24.99 mg QE g^−1^ of dry material). Within this group, accessions such as J543, J550 and J553 showed no significant difference (*p* > 0.05) in the TFC. American accessions had a relatively high TFC among the whole accession domain studied, ranging from 24.86 ± 2.90 to 216.67 ± 18.19 mg QE g^−1^ of dry material. Similar trends in variation were noticed in TFC contents in the accessions from other countries.

### 2.4. Screening of Antioxidant Potential in Sorghum Accessions

Sorghum has a wide geographical range, which results in variations in polyphenol content and biological activity. However, to date, the TPC, TFC, and antioxidant activity of sorghum accessions from the 15 different geographical regions have not been constructed. In this study, the antioxidant properties of 78 sorghum accessions collected from different countries and regions were estimated using DPPH and ABTS radical scavenging assays (Table 2). DPPH radical scavenging analysis revealed significant differences among the studied accessions. DPPH scavenging potential of the seed extracts from all the accessions ranged from 11.91 ± 4.83 to 1343.90 ± 81.02 µg mL^−1^, both figures observed in Indian accessions (I341 and I339, respectively). A wide variation range in DPPH scavenging activity was observed in the Korean accessions; at a concentration of 50 µg mL^−1^, their DPPH scavenging activity ranged from 23.81 ± 0.43 to 742.74 ± 18.23 µg mL^−1^. This is especially true for accession K384, which had an exceptionally high DPPH radical scavenging activity, with the highest RC50 value of 23.81 ± 0.43 µg mL^−1^, followed by accessions K386 and K666, with RC50 values of 38.67 ± 27.08 and 46.90 ± 5.99 µg mL^−1^, respectively. Similarly, a wide variation range (from 12.09 ± 3.14 to 408.18 ± 21.21 µg mL^−1^) in the antioxidant activity was observed within the American accessions. Notably, the antioxidant potential of accessions such as K384, U302, I341, and R505 was stronger than that of synthetic antioxidant compounds such as BHT (34.0 µg mL^−1^).

A large variation in the DPPH radical scavenging activity was also observed in the accessions from other countries. The results of the ABTS radical scavenging activity were similar to those of DPPH but showed some differences in the accessions. J547 showed the highest (69.99 ± 2.76 µg mL^−1^) ABTS radical scavenging activity, whereas I339 showed the lowest activity (3775.43 ± 311.91 µg mL^−1^). The ABTS value was higher than the DPPH value for most sorghum accessions, and a strong and positive correlation was observed between the two assays. A large variation (from 03.72 ± 0.40 to 28.98 ± 2.06 μmol TE g^−1^) in the ABTS scavenging activity was also observed in the Korean accessions; this variation could be due to differences in the ecological regions where the sorghum varieties were grown. A similar trend of variation in ABTS radical scavenging activity was observed for sorghum accessions collected from other countries.

### 2.5. Pearson’s Correlation Analysis between Antioxidant Activities, TPC, and TFC

**Figure 2:**
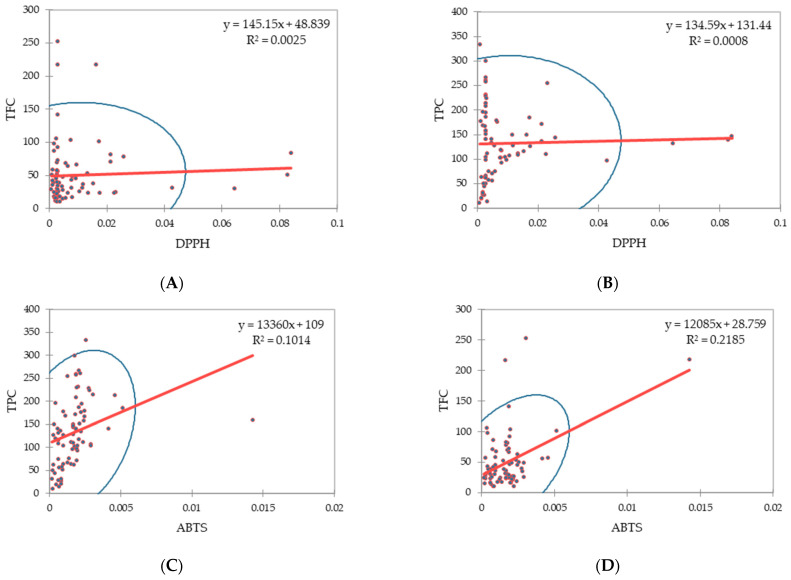
Correlation coefficients between the (**A**) DPPH and total flavonoid content, (**B**) DPPH and total phenolic content, (**C**) ABTS and total phenolic content, and (**D**) ABTS and total flavonoid content in selected *S. bicolor* accessions.

Pearson’s correlation analysis between antioxidant activity, TPC, and TFC was performed (Figure 2). The results showed a wide variation range in the correlation between antioxidant activities and TPC, as well as TFC. A weak positive association was observed between ABTS and TPC, as well as TFC (r = 0.1014 and r = 0.2185, respectively, *p* < 0.05) in the selected *S. bicolor* accessions, suggesting that both TPC and TFC contribute to the antioxidant activities of the sorghum accessions. Moreover, in this study, significant weak positive correlations were observed between DPPH scavenging and TPC, as well as TFC, in sorghum accessions. The significant variations in the total phenolic and flavonoid content in different accessions can be attributed to the acclimation of *S. bicolor* accessions to climatic conditions, which stresses the importance of their origins.

### 2.6. Principal Component Analysis (PCA)

**Figure 3:**
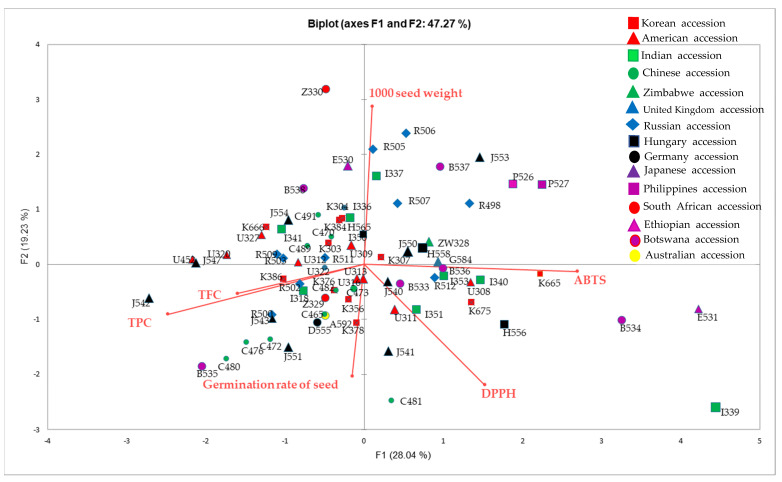
Principal component analysis (PCA) biplot constructed by plotting the PC1 (vertical axis) scores against the PC2 (horizontal axis) scores obtained from seed characteristics, antioxidant activity, and total phenolic and flavonoid content.

PCA is a multivariate technique that is useful for identifying patterns in produced data and elucidating possible relationships between parameters, which enables us to understand the association between characteristics and accessions. PCA was performed to reduce the dimensions of data and to elucidate the relationships among data items in 78 accessions of sorghum, collected from different geographical origins with distinct climatic conditions. The first and second principal components yielded 47.27% and 19.23% of the total variance, respectively (Figure 3). As shown in Figure 2, we identified accession groups using TPC, TFC, antioxidant activity, germination rate, and 1000-seed weight. Along axis 1 of the PCA analysis, the Russian accessions (R498, R505, and R5-6), Philippines accessions, (P526 and P527), Japanese accessions (J550 and J553), and Zimbabwe accession (ZW328) formed a clear group on the positive side and were associated with a higher 1000-seed weight. A majority of the Chinese accessions (C465, C472, C473, C476, C480, and C482) and Korean accessions (K356, K376, K378, and K386) contributed to the negative region of axis 1 (PC1) and were related to a higher seed germination rate and total phenolic and flavonoid content. Along axis 2 of the PCA analysis, four Indian accessions (I339, I340, I351, I353, and I-353), Japanese accession (J540 and J541), USA accessions (U311 and U308), Korean accessions (K665 and K675) and Botswana accessions (B533, B34 and B536) were primarily characterized by high antioxidant properties (DPPH and ABTS), indicating that accessions in this group have closely related genotypes. These results suggest that the antioxidant activity, TPC, and TFC profiles of sorghum accessions could be used as potential biomarkers for studying its biodiversity.

### 2.7. UPGMA Cluster Analysis 

**Figure 4:**
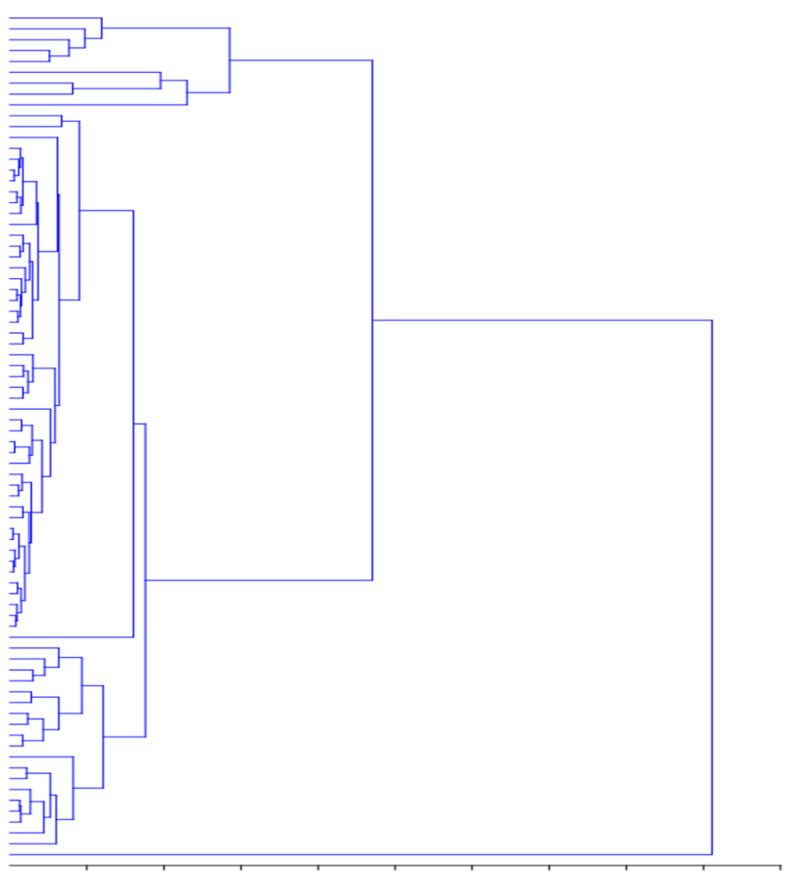
Dendrogram of morphological traits using UPGMA clustering in the selected accessions of *S. bicolor*.

A hierarchical cluster analysis (HCA) dendrogram was produced to measure the similarity between the clustered accessions, using Ward’s method in PAST software. Genetic divergence was observed mainly by UPGMA cluster analysis, with great dispersion of these accessions (Figure 4). All 78 accessions of sorghum were clustered into two main clusters. Cluster one was further divided into two subclusters: cluster 1A and cluster 1B. Subcluster 1A consisted of nine accessions, agglomerated mostly with low TPC and TFC. Subcluster 1B showed two distinct minor groups, represented by 1B-1 and 1B-2, in which the former minor group (1B-1) comprised 49 accessions, mainly from Korea, Russia, China, India, Japan, and the USA, and were mainly characterized by higher TPC and TFC. The latter minor group (1B-2) consisted of 19 accessions, mainly characterized by identical TPC, TFC, and antioxidant activity. Most accessions that collected a high level of diversity had been verified, although R502, of the A592 accession, had met under a single sub-grouping. The second group comprised a single accession (E531), which was mainly characterized by weaker antioxidant activity and lower amounts of TPC and TFC. The groups formed by the above methods were similar to those formed by principal component analysis.

## 3. Discussion

Rapid and uniform seed germination is an important factor that influences crop yield and seedling establishment [59]. This is the first report that assesses seed germination responses of a larger number of SB accessions. In the present study, high inter-accession variation was observed in the germination pattern of seed from various population of *S. bicolor.* Our results agree with the studies of Jung et al. [60], who showed a wide variation range (67–92%) in the germination percentage of Korean cultivars. Substantial variation in the germination rate of sorghum was also reported by Harris et al. [61], who observed a close association between seed size and seed germination rate in sorghum.

Understanding the grain weight (1000-seed weight) is required for the large-scale production of crops [62]. Seed weight is an important agronomic trait that may be helpful in breeding programs for the development of desired cultivars [63,64,65]. As expected, a significant variation in the 1000-seed weight and seed color was observed in the different accessions. This study is well correlated with the report of Alfieri et al. [62], who observed a wide 1000-seed weight range in sorghum genotypes. In this study, the correlation between 1000-seed weight and TPC was significant and positive in the Chinese and Japanese accessions, suggesting that phenolic compounds are important ingredients in increasing the 1000-seed weight; however, it was not significant in the Indian and Russian accessions. The contents of phenolics and flavonoids are highly influenced by various factors such as genetic factors, climatic conditions, a plant’s origin, a plant’s environment, altitudinal variation, and the duration of plant maturity [66,67,68]. In the present study, a wide range of variation was observed in the total phenolic and flavonoid contents in the *S. bicolor* accessions.

A wide variation range in the TPC of sorghum accessions has also been reported in the literature. Choi et al. [31] reported a significant variation in TPC of sorghum accessions, ranging from 1.56 ± 0.10 to 11.99 ± 2.01 e µg GAE/mg. Alferi et al. [62] had also observed a significant variation range in the TPC, ranging from 0.60 to 20.73 g GAE kg^−1^ dm^−1^. In another study, Dykes et al. [69] observed a similar variation range (1.35–37.73 mg GAE g^−1^ dm^−1^) in TPC of sorghum accessions. Using acetone solvent, increased TPC (ranging from 662 to 638 mg mg/GAE/100 g dry weight) was reported in sorghum cultivars. According to previous studies, the variation in TPC could be due to different factors, including extraction methods, solvent types, and ecological region, which could explain the wide variation range in the antioxidant activity and antioxidant accumulation patterns of sorghum accessions [70,71,72,73]. Large variations in the TFC was observed in the collected accessions. Total flavonoid content was significantly lower in Indian accessions than in accessions from other regions. High TFC variation was observed in the accessions collected from China, America, Japan, and Russia. In contrast, accessions including K384, U309, I339, ZW328, G584, R505, and Z330, originated from different geographic locations including Korea, America, India, Zimbabwe, United Kingdom, Russia, and South Africa, respectively, have similar trends of TFC accumulation with no significant difference at *p* > 0.05. The results indicate that these features are more genetically controlled as the environmental growing conditions were the same for all the tested accessions. High variability in the TPC content was also observed in the accessions originating from the same country with similar geographical landscape. For example, a high concentration and wide range in TPC was observed in the Chinese accessions. Within the group, the statistical analysis revealed a similar pattern of TPC content in the C481 and C489 accessions. Similarly, TPC content in the R505, R502 and R503 Russian accessions were not significantly different at *p* < 0.05. The variation between the sorghum accessions could be due to micro-evolutionary adaptations to different environments during the long-term evolutionary process. Moreover, it has been observed that the phenolic contents of plants can be influenced by various factors, including environmental conditions and temperature [74,75]. 

Previous studies reported that plants growing in extremely cold environmental conditions developed strong antioxidant properties to scavenge the ROS [76]. In the present study, the majority of accessions originating from Japan, Russia and Korea had a higher concentration of total phenolic content compared to the accessions from the Philippines and India. It is likely that *S. bicolor* growing in the colder environmental conditions of these countries might have developed strong antioxidants by accumulating more phytochemicals to scavenge the ROS. According to Zykova et al. [77], low temperature causes oxidative stress to the cells and causes elevation of ROS formation in the cells. Thus, to survive from the deleterious effect of ROS, plants accumulate larger amounts of phenolic compounds, which are efficient antioxidants [78]. Several studies observed elevated PAL activity (key enzymes in the biosynthesis of phenolic compounds increased during cold acclimation [79] and higher levels of phenolic compounds in the cells, which helps to reduce the freezing temperature of cellular components, thus improve the plant resistance to extreme colds [80].

Moreover, Jeon et al. [81] observed variations in the phenolic compounds and antioxidant properties owing to differences in the harvesting time of sorghum genotypes. In another study, the irradiance period of the growing sorghum genotypes influenced the phenolic content and antioxidant activity of sorghum plants [82,83,84]. Similarly, Przybylska-Balcerek et al. [85] observed variations in antioxidant activity and phenolic compound accumulation due to differences in the air temperature and precipitation rate during sorghum growth. In this study, since the environmental conditions experienced by the accessions were the same, variations in their TPC and TFC could be attributed to the genetic variation of individual sorghum accessions. A number of factors including climate, habitat, and genetic makeup influence the antioxidant properties of plants [86]. In the present study, we assessed the antioxidant potential of *S. bicolor* accessions using DPPH and ABTS radical scavenging assays. There was wide variation in the antioxidant activity within the accessions. In a similar study [31], a significant variation in DPPH in the range of 2.51 ± 0.28 to 40.18 ± 0.97 μmol TE/g was observed in the different sorghum accessions; their range varies largely with that of the present study, and this difference could be due to differences in the ecological regions of the collected sorghum. The antioxidant data obtained by the two assays were notably different, but were highly correlated, which was also corroborated by the results of Dykes et al. [87]. A wide variation in the antioxidant activity (DPPH and ABTS) of sorghum accessions was also reported by Awika et al. [17] and Contrera [88] in sorghum genotypes grown in different ecological regions of Texas, USA. Phytochemicals conduct various biological activities, including biochemical and pharmacological properties [89], and have a high polyphenol antioxidant potential [90]. Several studies have reported the presence of various bioactive compounds, including flavonoids (flavanonols and flavan-3-ol derivatives), phenylpropane glycerides, dicaffeoyl spermidine, condensed tannins, flavanones, and flavonols, which act as radical scavengers in sorghum [25,91,92]. 

It has been argued that the antioxidant properties and biosynthesis of phenolic compounds are affected by agronomic conditions, including climate patterns and geographical origin of the region [67]. Additionally, other factors, such as antioxidant assays and solvent types used in the assays, also contribute to the reported variations in plant antioxidant properties [93]. Further, agronomic manipulation, such as proper irrigation, increases the antioxidant activities of sorghum genotypes [71]. In the present study, a wide range of variation in antioxidant properties was observed within populations originating from same region. This trend was more visible in the accessions collected from India (ranging from 11.91µg mL^−1^ in I341 to 1343.92 µg mL^−1^ in I339), China (ranging from 46.90 µg mL^−1^ in C470 to 838.02 µg mL^−1^ in C481), and Korea (ranging from 23.81 µg mL^−1^ in K384 to 742.74 µg mL^−1^ in K975). In contrast, sorghum accessions including K304, U316, I318, C465, R500, D55, J547, P527, Z330, B535, and A592 originated from different countries but revealed similar trends of antioxidant properties and did not vary significantly (*p* > 0.05) in terms of DPPH radical scavenging activity. Higher variability in the antioxidant properties within the accessions from the same region or across entire accessions could be due to the different genetic features of individual accessions under the same environmental growing conditions. In this study, some sorghum accessions, such as I341, contained high TPC and TFC and showed strong DPPH radical scavenging activities, whereas the I339 accession contained low TPC and TFC and showed weak antioxidant activities. Further, some sorghum accessions, such as I341, contained high TPC and TFC and showed strong DPPH radical scavenging activities, indicating that phenolic compounds significantly contributed to antioxidant activity in the sorghum accessions. However, accessions such as BWA534 and BWA536 recorded similar TPC and TFC but varied significantly in DPPH and ABTS radical scavenging properties. The results further indicate that different accessions have varying phenolic compound components, which could be the reason for the variability of the antioxidant activities of sorghum accessions. The J542 sorghum accession, which contains high TPC and TFC, exhibited weak DPPH and ABTS radical scavenging activities. These variations indicate that phytochemicals other than the phenolic compounds may contribute to the antioxidant properties of sorghum accessions. 

In this study, the white-colored accession (E531) showed low TPC and TFC and exhibited the weakest antioxidant activity (ABTS) among all the studied sorghum accessions. This was in agreement with the results of Wu et al. [71], who observed a low antioxidant activity in white sorghum. Moreover, Chung et al. [94], Awika and Rooney [4], and Dykes et al. [87] observed wide variation in the antioxidant compounds, such as tannins, in the sorghum accessions. John et al. [95] observed the presence of large amounts of carotenoids, phenolic acids, flavonoids, and tannins [8,96,97] in sorghum accessions, contributing to antioxidant activity. Moreover, in a similar study, high concentrations of phenolic acids, flavonoids, and carotenoids in sorghum grains were observed [85,98,99,100]. 

## 4. Materials and Methods

### 4.1. Chemicals

All solvents used in this study were of analytical grade. Methanol was obtained from Baker (Phillipsburg, NJ, USA). Sodium carbonate, gallic acid, potassium acetate, aluminum nitrate, and aluminum chloride were obtained from Sigma-Aldrich Chemical Co. (St. Louis, MO, USA). 1,1-Diphenyl-2-picrylhydrazyl (DPPH), 2,2-azino-bis-3-ethylbenzthiazoline-6-sulphonic acid (ABTS^+^), Folin–Ciocalteu reagent, quercetin, and tert-butyl-4-hydroxy toluene (BHT) were obtained from Sigma Chemical Co. (St. Louis, MO, USA). Ultrapure distilled water was obtained from the Zeneer power 1 system (Human Corporation, Seoul, Korea).

### 4.2. Cultivation of S. italica

For morphological characterization, TPC, TFC, and antioxidant activity evaluation, 78 sorghum accessions were sown at the experimental farmland of Kangwon National University, at Chuncheon, Kangwon-Do, South Korea, located at 20°45′ S, 42°51′ W with an average altitude of 650 m, on the first week of June and harvested in mid-November of 2018, 2019 and 2020. These *S. italica* accessions originated from Korea, U.S.A., India, China, Zimbabwe, United Kingdom, Russia, Hungary, Germany, Japan, Philippines, South Africa, Ethiopia, Botswana, and Australia. All the experiments were carried out in a completely randomized block design. The experimental plots consisted of rows of 80 m in length, spaced 1.5 m apart with 1m between the planted seedlings. Seeds of each *S. italica* accession were sown in each plot. The mean minimum and maximum field temperatures during the cultivation period were 20 °C and 35 °C, respectively, with an approximate rainfall rate of 200 nm. The sandy loam texture of the experimental field was maintained at a pH of 6.1. The cultivated field was irrigated regularly once weekly by installing a drip-irrigation system. The recommended doses of compound chemical fertilizers were applied to the experimental field (Nitrogen: Phosphorus: Potassium = 15%:15%:15%) at a rate of 120 kg ha−1 before the seedlings were planted. Landscape fabric was used as a weed blocker to cover space between the rows. Weeds were manually removed regularly during the seedling growth. Diseases were controlled by recommended pesticides. Morphological traits such as seed colour, 1000-seed weight, days to flower and plant height were recorded from ten selected plants of each accession, prior to harvesting the plants.

### 4.3. Germination Test of Sorghum Accessions

Seeds of the 15 sorghum accessions were kindly provided by RDA, South Korea, in 2018, 2019, and 2020. The collected seeds were stored in the dark at 4 °C until further use. Fifty seeds from each accession were placed in a Petri dish (SPL Life Sciences Co., Ltd., Pocheon, Korea) on wet filter paper. Sterile deionized water was used to wet the filter paper. Three replicates of each treatment were performed, and all treatments were maintained at 25 ± 1 °C for 40 days in a growth chamber with an 8:16 light and dark cycle. Germination rate was calculated as the percentage of seeds that germinated out of the total number of seeds.

### 4.4. Plant Material Extractions

Approximately 10 g of the seeds from each accession were finely ground using a homogenizer. The powdered samples were mixed with 80% methanol at room temperature (25 °C) for 24 h. The mixture was then filtered to remove debris using Whatman No.42 filter paper. The obtained extracts were then evaporated at 40 °C in a rotary evaporator (Eyela, SB-1300, Shanghai Eyela Co. Ltd., Shanghai, China). The extracts were suspended in distilled water and used for further analysis.

### 4.5. Determination of Total Phenolic Compounds (TPC)

TPC of individual sorghum accession seed extracts was determined using the Folin–Ciocalteu spectrophotometric method (Singleton et al., 1999, as described elsewhere [101]). TPC estimation was carried out in triplicate in 10 mL test tubes. Initially, 100 μL of diluted seed extract (1 mg mL^−1^) was mixed with 50 μL of Folin–Ciocalteu reagent (1 M). Subsequently, 1.85 mL of distilled water was added to the mixture and allowed to stand in the dark at room temperature (25 °C) for 5 min. After incubation, 1.0 mL of Na_2_CO_3_ (20% *w/v*) was added to the mixture and shaken slowly with intermittent agitation. After 1 h, the absorbance value of each seed extract was recorded using a spectrophotometer (Jasco V530 UV-VIS spectrophotometer, Tokyo, Japan) at 725 nm wavelength against the blank, without extracts. The results were expressed as gallic acid equivalents (GAE) per gram of dry sample.

### 4.6. Determination of Total Flavonoid Content (TFC)

The TFC of the seed extracts of sorghum accessions was quantified using the colorimetric method proposed by Moreno et al. [102]. Briefly, 250 μL of seed extract sample (1 mg mL^−1^) was mixed with 100 µL of 10% AlNO_3_ and 100 µL of potassium acetate (1 M), and 4.3 mL of 80% ethanol was added to the mixture to achieve a final volume of 5 mL. The reaction mixture was shaken well and allowed to react for 10 min. The absorbance value of the mixture was recorded in triplicate at 410 nm wavelength using a spectrophotometer. The standard curve was made of quercetin. The TFC of the seed extracts of each accession was expressed in terms of quercetin equivalent (QE) per gram of dry sample.

### 4.7. Antioxidant Activity

#### 4.7.1. Evaluation of DPPH Radical Scavenging Assay

DPPH radical scavenging capacity was determined as previously described [103], with some modifications. Initially, 200 µL aliquots of the seed extract at different concentrations (ranging from 0.05 to 10 mg mL^−1^) were mixed with 4.5 mL of DPPH methanolic solution (0.004% in methanol). The mixture was shaken thoroughly and incubated at room temperature (25 °C) for 40 min in the dark. The absorbance of the mixture was measured using a spectrophotometer at 517 nm with methanol as the blank. The DPPH radical scavenging activity of seed extracts of SB accessions was calculated as follows:

DPPH scavenging activity = (Abs_control_ − Abs_sample_)/Abs_control_

where Abs_control_ indicates the absorbance value of the reaction mixture without seed extracts. Abs_sample_ represents the absorbance of the reaction mixture with SB seed extracts. 

#### 4.7.2. Evaluation of ABTS+ Assay

The ABTS radical scavenging activity assay was carried out according to a previously described method [104] with modifications. Briefly, the oxidation reaction mixture was prepared by mixing 7.4 mM·L^−1^ ABTS with 2.6 mM·L^−1^ potassium persulfate (final concentration). Subsequently, the reaction mixture was incubated at room temperature (25 °C) for 12 h in the dark. The mixture was diluted with methanol (80%), and the absorbance of the mixture was measured at 734 nm using a spectrophotometer. Trolox (acid 6-hydroxy-2,5,7,8-tetramethylchroman-2-carboxylic) was used as a standard to construct the standard calibration curve. The results were expressed as trolox equivalents (TE) per gram dry weight (μmol TE·g^−1^ DW). The ABTS radical scavenging activity of seed extracts of SB accessions was calculated as follows:

ABTS scavenging activity = (Abs_control_ − Abs_sample_)/Abs_control_ × 100

where Abs_control_ indicates the absorbance of the ABTS solution without seed extracts. Abs_sample_ is the absorbance of the ABTS solution in the seed extract sample. 

### 4.8. Statistical Analysis

Data were obtained in triplicate and reported as the mean ± standard deviation. For the statistical analysis, one-way analysis of variance (ANOVA) was used, followed by Duncan’s multiple comparison tests at *p* < 0.05 and *p* < 0.01. Principal component analysis (PCA) of morphological traits and phenolic compounds was conducted and Pearson’s correlation coefficient between TPC, TFC, and antioxidant activity of sorghum accessions was determined using SPSS version 20 (SPSS, 2011). 

## 5. Conclusions

This study is the first to report the seed characteristics, antioxidant activity, and polyphenol contents of 78 sorghum accessions collected from 15 countries. Pearson’s correlation revealed a wide variation range in these parameters, which may contribute significantly to their antioxidant activity. TPC, TFC, and antioxidant properties of the accessions were not related to the geographical origins of the samples. Among the accessions characterized by DPPH analysis, K334, U312, I341, and R505 presented higher antioxidant values with relatively high TPCs and suitable 1000-seed weights and germination rates, thus acting as potentially useful sorghum accessions for large-scale food production and as sources of antioxidant compounds. Improved accessions with high levels of phenolic constituents and higher antioxidant properties are of great interest to the producers and consumers of nutraceutical supplements. Further studies are required to isolate and identify phytochemicals from diverse accessions to determine the major contributors to antioxidant activity. This study presented the variability in agronomic traits, antioxidant activity, TPC, and TFC among the 78 sorghum accessions, which can be used as baseline data in breeding programs and germplasm management to develop sorghum with beneficial traits and improved nutritional value, for potential positive effects on human health.

## Figures and Tables

**Table 1:** Germination rate and 1000-seed weight in *S. bicolor* accessions with different origins.

Country	Accession	1000-Seed Weight (g) ^1^	Germination Rate (%)
Korea	K303	18.00 ± 1.00 ^g^	74.00 ± 3.00 ^k^
	K304	23.00 ± 1.10 ^l^	52.80 ± 2.00 ^f^
	K307	20.00 ± 1.50 ^i^	98.00 ± 2.00 ^bb^
	K384	23.20 ± 1.00 ^l^	95.70 ± 4.00 ^y^
	K665	26.00 ± 1.30 ^n^	98.00 ± 2.10 ^bb^
	K675	18.00 ± 1.50 ^g^	66.50 ± 2.00 ^i^
	K378	11.00 ± 0.90 ^b^	100.0 ± 0.0 ^dd^
	K386	15.00 ± 0.70 ^d^	84.80 ± 2.00 ^p^
	K356	16.07 ± 1.00 ^e^	82.00 ± 1.50 ^n^
	K376	14.05 ± 1.11 ^c^	94.00 ± 3.00 ^x^
	K666	20.70 ± 1.50 ^i^	68.50 ± 2.00 ^j^
USA	U309	21.00 ± 1.00 ^j^	92.90 ± 4.00 ^w^
	U311	15.03 ± 1.00 ^d^	90.50 ± 3.00 ^u^
	U312	17.05 ± 1.50 ^f^	90.70 ± 2.00 ^u^
	U313	19.00 ± 2.00 ^h^	90.40 ± 3.00 ^u^
	U320	20.00 ± 2.00 ^i^	82.70 ± 2.00 ^n^
	U327	21.06 ± 2.50 ^j^	72.90 ± 3.00 ^k^
	U322	19.03 ± 1.50 ^h^	98.40 ± 2.00 ^bb^
	U452	22.90 ± 2.00 ^k^	86.90 ± 1.50 ^q^
	U308	18.00 ± 1.90 ^g^	74.40 ± 3.00 ^k^
	U316	17.00 ± 1.00 ^f^	72.00 ± 2.00 ^k^
India	I318	21.00 ± 1.00 ^j^	92.00 ± 3.00 ^v^
	I336	24.00 ± 1.11 ^m^	86.00 ± 3.00 ^q^
	I337	17.00 ± 1.50 ^f^	8.00 ± 0.50 ^a^
	I339	16.00 ± 1.00 ^e^	86.00 ± 3.00 ^q^
	I340	18.00 ± 1.30 ^g^	86.00 ± 3.00 ^q^
	I341	10.00 ± 0.80 ^a^	18.90 ± 1.00 ^c^
	I353	20.00 ± 1.10 ^i^	72.60 ± 2.00 ^k^
	I350	22.00 ± 1.50 ^k^	92.40 ± 4.00 ^v^
	I351	15.00 ± 1.10 ^d^	94.30 ± 4.00 ^x^
China	C465	17.20 ± 1.500 ^f^	96.00 ± 3.00 ^z^
	C470	22.00 ± 2.00 ^k^	88.80 ± 4.90 ^r^
	C481	10.00 ± 0.80 ^a^	75.90 ± 5.00 ^l^
	C482	17.00 ± 1.00 ^f^	94.00 ± 2.00 ^x^
	C491	26.00 ± 2.00 ^n^	96.50 ± 3.00 ^z^
	C489	23.90 ± 2.90 ^l^	97.40 ± 2.10 ^aa^
	C472	16.00 ± 1.00 ^e^	99.00 ± 1.00 ^cc^
	C473	19.00 ± 1.20 ^h^	92.99 ± 4.00 ^w^
	C480	10.00 ± 0.90 ^a^	72.00 ± 5.00 ^k^
	C476	14.00 ± 1.00 ^c^	86.00 ± 5.00 ^q^
Zimbabwe	ZW328	17.00 ± 1.00 ^f^	62.00 ± 3.00 ^h^
United Kingdom	G584	22.00 ± 2.00 ^k^	94.00 ± 3.00 ^x^
Russia	R498	28.00 ± 2.90 ^p^	97.80 ± 2.00 ^aa^
	R500	19.00 ± 1.90 ^h^	98.00 ± 1.50 ^bb^
	R505	34.90 ± 2.50 ^t^	95.90 ± 4.00 ^y^
	R506	22.00 ± 2.00 ^k^	10.00 ± 0.50 ^b^
	R507	30.00 ± 3.00 ^r^	91.90 ± 4.00 ^v^
	R511	18.00 ± 1.50 ^g^	82.80 ± 3.00 ^n^
	R502	21.00 ± 2.00 ^j^	86.60 ± 5.00 ^q^
	R503	27.00 ± 1.50 ^o^	96.90 ± 2.00 ^z^
	R509	22.00 ± 2.00 ^k^	88.70 ± 4.00 ^r^
	R512	18.00 ± 2.50 ^g^	97.60 ± 2.00 ^aa^
Hungary	H556	15.40 ± 1.55 ^d^	72.00 ± 4.00 ^k^
	H558	22.50 ± 2.00 ^k^	96.00 ± 2.20 ^z^
	H565	29.60 ± 3.00 ^q^	90.00 ± 5.50 ^u^
Germany	D555	16.00 ± 1.00 ^e^	98.00 ± 1.10 ^bb^
Japan	J540	23.00 ± 1.50 ^l^	83.00 ± 4.90 ^o^
	J547	26.00 ± 2.00 ^n^	82.00 ± 5.00 ^n^
	J551	28.00 ± 2.00 ^p^	96.00 ± 2.00 ^z^
	J541	14.00 ± 1.50 ^c^	75.00 ± 5.00 ^l^
	J542	23.00 ± 1.50 ^l^	83.00 ± 4.00 ^o^
	J543	19.00 ± 1.00 ^h^	100.00 ± 0.0 ^dd^
	J550	23.80 ± 2.00 ^l^	97.00 ± 2.00 ^aa^
	J553	32.00 ± 1.00 ^s^	74.00 ± 2.00 ^k^
	J554	29.00 ± 2.00 ^q^	80.00 ±4.00 ^m^
Philippines	P526	26.00 ± 1.50 ^n^	42.90 ± 3.00 ^e^
	P527	27.63 ± 2.00 ^o^	54.89 ± 4.60 ^g^
South Africa	Z329	21.00 ± 1.50 ^j^	98.00 ±2.00 ^bb^
	Z330	35.80 ± 2.00 ^u^	10.00 ± 0.50 ^b^
Ethiopia	E530	23.00 ± 2.90 ^l^	26.70 ± 2.00 ^d^
	E531	19.00 ± 1.00 ^h^	94.00 ± 3.00 ^x^
Botswana	B533	18.00 ± 1.40 ^g^	98.00 ± 1.50 ^bb^
	B534	16.00 ± 1.50 ^e^	96.00 ± 3.00 ^z^
	B535	12.00 ± 1.00 ^b^	86.40 ±4.50 ^q^
	B536	17.00 ± 1.50 ^f^	88.00 ± 4.60 ^r^
	B537	28.13 ± 1.40 ^p^	66.00 ± 3.00 ^i^
	B538	30.80 ± 3.00 ^r^	89.00± 4.00 ^t^
Australia	A592	18.00 ± 1.00 ^g^	100.00 ± 0.0 ^dd^

^1^ Data with the same letter in a row did not differ significantly according to Duncan’s multiple comparison test (*p* < 0.05). Mean values within a column with the same lowercase letters were not significantly different (*p* < 0.05) according to Duncan’s multiple comparison test.

**Table 2:** Antioxidant activity, total phenolic and total flavonoid content of the selected *S. bicolor* accessions.

Country ^1^	Accession	DPPH (µg mL^−1^)	ABTS (µg mL^−1^)	Total Flavonoid Content (mg QE g^−1^)	Total Phenol Content (mg GAE g^−1^)
Korea	K303	83.98 ± 17.97 ^h^	586.85 ± 3.37 ^y^	56.35 ± 3.53 ^q^	127.63 ± 1.76 ^t^
	K304	359.06 ± 6.19 ^xy^	413.53 ± 7.72 ^lm^	19.02 ± 0.27 ^cd^	156.91 ± 1.09 ^y^
	K307	65.21 ± 2.09 ^f^	1605.56 ± 10.74 ^tt^	37.89 ± 3.30 ^k^	115.65 ± 1.47 ^r^
	K384	23.81 ± 0.43 ^b^	609.07 ± 6.34 ^bb^	30.98 ± 1.97 ^g^	96.01 ± 1.95 ^o^
	K665	667.98 ± 11.72 ^hh^	2097.07 ± 5.04 ^ww^	38.75 ± 2.66 ^l^	18.98 ± 1.68 ^c^
	K975	742.74 ± 18.23 ^ii^	1011.19 ± 16.50 ^ii^	35.52 ± 1.03 ^j^	62.86 ± 17.53 ^j^
	K378	107.37 ± 7.02 ^kl^	1025.35 ± 18.88 ^jj^	45.05 ± 2.09 ^mn^	104.12 ± 1.31 ^pq^
	K386	38.67 ± 7.08 ^c^	594.21 ± 5.84 ^z^	77.48 ± 4.61 ^w^	144.12 ± 1.83 ^x^
	K356	350.18 ± 11.21 ^wx^	469.40 ± 3.70 ^pq^	21.30 ± 1.46 ^d^	150.60 ± 1.41 ^xy^
	K376	94.9 ± 4.06 ^j^	339.58 ± 12.57 ^f^	25.37 ± 1.42 ^ef^	107.00 ± 1.27 ^pq^
	K666	46.9 ± 5.99 ^de^	548.63 ± 7.30 ^w^	81.13 ± 4.57 ^x^	171.50 ± 3.80 ^aa^
USA	U309	127.79 ± 3.05 ^lm^	591.70 ± 3.96 ^yz^	30.47 ± 1.88 ^g^	101.95 ± 1.16 ^p^
	U311	375.72 ± 17.60 ^z^	659.69 ± 2.85 ^dd^	51.82 ± 2.88 ^o^	62.77 ± 1.87 ^j^
	U312	12.09 ± 3.14 ^a^	529.13 ± 7.01 ^v^	50.32 ± 2.18 ^no^	139.25 ± 1.68 ^v^
	U313	293.95 ± 8.06 ^t^	644.95 ± 8.52 ^cc^	38.75 ± 1.31 ^l^	111.68 ± 1.56 ^q^
	U320	57.66 ± 3.3 ^e^	194.16 ± 8.95 ^b^	100.88 ± 1.85 ^cc^	184.66 ± 1.80 ^cc^
	U327	43.23 ± 2.29 ^d^	774.55 ± 14.86 ^ff^	23.85 ± 1.71 ^e^	255.11 ± 1.77 ^ii^
	U322	103.78 ± 1.47 ^k^	531.96 ± 11.41 ^v^	66.13 ± 4.42 ^s^	97.45 ± 3.13 ^o^
	U452	61.44 ± 3.71 ^ef^	601.44 ± 3.20 ^aa^	216.67 ± 8.19 ^gg^	149.88 ± 1.80 ^xy^
	U308	408.18 ± 5.21 ^bb^	2524.47 ± 30.70 ^yy^	105.71 ± 6.46 ^ee^	43.94 ± 1.41 ^g^
	U316	345.00 ± 9.34 ^wx^	536.50 ± 11.16 ^w^	24.86 ± 2.90 ^ef^	140.24 ± 1.27 ^vw^
India	I318	355.35 ± 8.08 ^x^	492.8 ± 5.57 ^r^	15.84 ± 0.24 ^bc^	230.87 ± 1.13 ^gg^
	I336	44.22 ± 2.49 ^d^	588.65 ± 16.05 ^y^	23.46 ± 0.11 ^e^	109.52 ± 1.41 ^q^
	I337	206.93 ± 9.35 ^q^	512.08 ± 14.19 ^u^	47.09 ± 2.13 ^n^	70.96 ± 1.31 ^l^
	I339	1343.90 ± 8.02 ^mm^	3775.43 ± 11.91 ^ccc^	28.31 ± 1.85 ^fg^	9.97 ± 1.00 ^a^
	I340	430.53 ± 10.46 ^cc^	1197.80 ± 20.82 ^ll^	10.98 ± 1.09 ^a^	26.01 ± 1.16 ^d^
	I341	11.91 ± 0.83 ^a^	592.20 ± 2.11 ^yz^	83.21 ± 6.06 ^y^	146.55 ± 1.49 ^x^
	I353	544.25 ± 7.09 ^ff^	1220.9 ± 7.65 ^nn^	86.55 ± 2.32 ^z^	31.77 ± 1.83 ^f^
	I350	124.86 ± 5.55 ^l^	498.94 ± 10.07 ^s^	16.72 ± 0.53 ^bc^	92.32 ± 1.31 ^n^
	I351	366.6 ± 9.15 ^y^	780.79 ± 9.93 ^gg^	22.36 ± 3.02 ^de^	66.28 ± 1.62 ^k^
China	C465	349.87 ± 7.94 ^wx^	443.08 ± 9.48 ^n^	10.35 ± 0.57 ^a^	194.84 ± 1.72 ^dd^
	C470	46.90 ± 5.99 ^de^	1299.65 ± 20.71 ^oo^	70.42 ± 4.27 ^u^	135.47 ± 1.63 ^uv^
	C481	838.02 ± 26.48 ^kk^	1011.17 ± 6.28 ^ii^	57.99 ± 2.87 ^q^	177.27 ± 1.24 ^bb^
	C482	174.5 ± 5.21 ^p^	988.66 ± 16.75 ^hh^	67.94 ± 2.17 ^s^	127.18 ± 1.87 ^t^
	C491	74.71 ± 6.50 ^g^	419.10 ± 8.51 ^m^	53.26 ± 2.48 ^p^	110.78 ± 1.27 ^q^
	C489	152.57 ± 5.04 n	452.53 ± 1.96 ^o^	23.38 ± 1.19 e	175.83 ± 5.82 ^bb^
	C472	346.85 ± 9.71 ^wx^	480.08 ± 9.02 ^qr^	25.32 ± 1.79 ^ef^	266.82 ± 1.95 ^kk^
	C473	353.10 ± 7.41 ^x^	344.85 ± 10.54 ^g^	49.14 ± 1.50 ^no^	103.49 ± 1.27 ^pq^
	C480	349.81 ± 5.27 ^wx^	563.03 ± 3.31 ^x^	69.39 ± 1.16 ^st^	299.52 ± 2.72 ^ll^
	C476	344.86 ± 5.48 ^w^	524.67 ± 12.73 ^v^	72.50 ± 4.74 ^uv^	259.52 ± 2.71 ^jj^
Zimbabwe	ZW328	87.09 ± 6.43 ^i^	2780.69 ± 17.73 ^zz^	30.81 ± 1.31 ^g^	149.61 ± 1.63 ^xy^
United Kingdom	G584	364.16 ± 7.12 ^y^	1078.97 ± 41.26 ^kk^	30.25 ± 1.43 ^g^	50.60 ± 1.41 ^h^
Russia	R498	57.14 ± 31.68 ^e^	3284.10 ± 30.91 ^aaa^	23.36 ± 1.29 ^e^	125.92 ± 1.47 ^t^
	R500	355.07 ± 5.85 ^x^	217.87 ± 11.95 ^c^	27.06 ± 2.29 ^f^	213.31 ± 1.74 ^ff^
	R505	15.52 ± 1.44 ^ab^	1551.00 ± 13.31 ^rr^	30.17 ± 1.88 ^g^	131.05 ± 1.54 ^gg^
	R506	73.78 ± 4.64 ^g^	1543.58 ± 5.26 ^qq^	22.97 ± 1.29 ^de^	107.90 ± 1.33 ^pq^
	R507	217.77 ± 4.08 ^r^	1724.58 ± 9.09 ^uu^	32.89 ± 1.81 ^h^	140.33 ± 1.62 ^vw^
	R511	125.36 ± 7.70 ^l^	477.77 ± 6.44 ^q^	48.85 ± 1.41 ^n^	117.81 ± 2.21 ^rs^
	R502	349.33 ± 2.93 ^wx^	514.64 ± 12.75 ^uv^	24.34 ± 1.74 ^ef^	229.52 ± 1.62 ^gg^
	R503	351.00 ± 12.50 ^wx^	359.94 ± 4.10 ^h^	39.86 ± 2.03 l^m^	228.17 ± 1.54 ^gg^
	R509	156.34 ± 6.73 ^no^	400.78 ± 11.26 ^k^	63.34 ± 1.46 ^r^	178.80 ± 1.16 ^bb^
	R512	258.02 ± 10.70 ^s^	1128.02 ± 21.71 ^ll^	10.17 ± 0.91 ^a^	57.90 ± 0.87 ^i^
Hungary	H556	765.75 ± 10.46 ^jj^	1200.10 ± 12.81 ^mm^	71.62 ± 2.83 ^u^	20.69 ± 1.41 ^cd^
	H558	128.43 ± 5.81 l^m^	2379.52 ± 16.38 ^xx^	43.43 ± 4.23 ^m^	118.98 ± 2.25 ^rs^
	H565	343.86 ± 6.59 ^w^	511.37 ± 11.73 ^u^	92.48 ± 4.08 ^aa^	97.72 ± 1.09 ^o^
Germany	D555	346.27 ± 9.44 ^wx^	239.14 ± 6.11 ^d^	56.25 ± 0.42 ^q^	141.14 ± 1.22 ^vw^
Japan	J540	556.10 ± 14.06 ^gg^	595.17 ± 15.20 ^z^	17.33 ± 0.13 ^c^	168.26 ± 1.33 ^z^
	J547	346.48 ± 10.19 ^wx^	69.99 ± 2.76 ^a^	217.50 ± 11.77 ^hh^	158.71 ± 1.09 ^yz^
	J551	994.01 ± 10.31 ^ll^	386.62 ± 3.12 ^hi^	50.17 ± 3.82 ^no^	333.58 ± 1.49 ^mm^
	J541	557.48 ± 10.72 ^gg^	2379.52 ± 16.38 ^xx^	98.24 ± 4.99 ^bb^	196.37 ± 2.60 ^dd^
	J542	344.68 ± 11.11 ^w^	326.91 ± 8.60 ^e^	252.67 ± 24.99 ^ii^	213.85 ± 0.68 ^ff^
	J543	342.59 ± 6.76 ^w^	462.01 ± 14.35 ^p^	26.64 ± 1.38 ^f^	260.60 ± 2.25 ^jj^
	J550	344.81 ± 11.57 ^w^	585.24 ± 12.88 ^y^	24.78 ± 3.42 ^ef^	62.32 ± 0.16 ^j^
	J553	302.97 ± 13.68 ^u^	1403.41 ± 9.23 ^pp^	25.00 ± 9..84 ^ef^	13.94 ± 0.31 ^b^
	J554	320.72 ± 12.33 ^v^	355.24 ± 3.26 ^h^	34.32 ± 7.61 ^i^	224.30 ± 2.34 ^gg^
Philippines	P526	512.91 ± 10.72 ^ee^	1584.08 ± 5.43 ^gg^	16.03 ± 4.00 ^bc^	27.90 ± 0.68 ^de^
	P527	356.90 ± 5.41 ^x^	3551.25 ± 40.29 ^bbb^	57.40 ± 29.08 ^q^	62.05 ± 0.41 ^j^
South Africa	Z329	400.39 ± 7.010 ^aa^	405.33 ± 11.98 ^l^	44.93 ± 0.56 ^m^	166.91 ± 0.68 ^z^
	Z330	346.59 ± 4.93 ^wx^	524.71 ± 6.50 ^v^	28.80 ± 0.29 ^fg^	207.99 ± 4.60 ^ee^
Ethiopia	E530	215.37 ± 5.66 ^r^	450.24 ± 1.97 ^o^	27.18 ± 5.41 ^f^	133.58 ± 0.87 ^u^
	E531	562.18 ± 7.49 ^gg^	5517.65 ± 9.35 ^eee^	24.78 ± 1.36 ef	30.51 ± 0.00 ^f^
Botswana	B533	268.54 ± 7.57 ^s^	717.82 ± 8.81 ^ee^	17.72 ± 0.15 ^c^	75.11 ± 0.00 ^m^
	B534	467.05 ± 9.60 ^dd^	4352.41 ± 8.11 ^ddd^	15.27 ± 0.64 ^bc^	50.15 ± 0.41 ^h^
	B535	347.75 ± 8.13 ^wx^	528.56 ± 4.23 ^v^	141.30 ± 82.31 ^ff^	258.08 ± 2.21 ^jj^
	B536	206.69 ± 5.13 ^q^	1429.32 ± 10.36 ^pp^	14.27 ± 0.19 ^b^	56.28 ± 1.49 ^i^
	B537	173.08 ± 8.87 ^p^	1558.49 ± 8.21 ^gg^	15.74 ± 2.82 ^bc^	74.12 ± 0.41 ^m^
	B538	131.57 ± 6.65 ^m^	500.47 ± 9.55 ^t^	103.41 ± 43.76 ^dd^	103.04 ± 0.87 ^pq^
Australia	A592	351.50 ± 6.72 ^wx^	480.43 ± 4.90 ^qr^	21.74 ± 0.87 ^d^	186.55 ± 1.33^cc^

^1^ Data with the same letter in a column did not differ significantly according to Duncan’s multiple comparison test (*p* < 0.05). Mean values within a column with the same lowercase letters were not significantly different (*p* < 0.05) according to Duncan’s multiple comparison test.

## Data Availability

Not applicable.

## Sample Availability

Samples of the compounds are available from the authors.

## References

[1] Queiroz V.A.V., Moraes E.A., Schaffert R.E., Moreira A.V., Ribeiro S.M.R., Martino H.S.D. (2011). Potencial funcional e tecnologia de processamento do sorgo [*Sorghum bicolor* (L.) Moench], na alimentação humana. Rev. Bras. Milho Sorgo.

[2] FAOSTAT. http://faostat.fao.org/.

[3] Awika J.M., Rooney L.W. (2004). Sorghum phytochemical and their potential impact on human health. Phytochemistry.

[4] Dykes L., Rooney L.W. (2007). Phenolic compounds in cereal grains and their health benefits. Cereal Food World.

[5] De Morais Cardoso L., Montini T.A., Pinheiro S.S., Pinheiro-Sant’Ana H.M., Martino H.S.D., Moreira A.V.B. (2014). Effects of processing with dry heat and wet heat on the antioxidant profile of sorghum. Food Chem..

[6] Chávez D.W.H., Ascheri J.L.R., Carvalho C.W.P., Godoy R.L.O., Pacheco S. (2017). Sorghum and roasted coffee blends as a novel extruded product: Bioactive compounds and antioxidant capacity. J. Funct. Foods.

[7] Llopart E.E., Drago S.R. (2016). Physicochemical properties of sorghum and technological aptitude for popping nutritional changes after popping. LWT Food Sci. Technol..

[8] Stefoska-Needham A., Beck E.J., Johnson S.K., Tapsell L.C. (2015). Sorghum: An underutilized cereal whole grain with the potential to assist in the prevention of chronic disease. Food Rev. Int..

[9] Sanchez A.C., Subudhi P.K., Rosenow D.T., Nguyen H.T. (2002). Mapping QTLs associated with drought resistance in sorghum (*Sorghum bicolor* L. Moench). Plant Mol. Biol..

[10] Sanchez D.A. (2003). White Food-Type Sorghum in Direct-Expansion Extrusion Applications Master of Science. Master’s Thesis.

[11] Belton P.S., Taylor J.R.N. (2004). Sorghum and millets: Protein sources for Africa. Trends Food Sci. Technol..

[12] Dicko M.H., Gruppen H., Traore A.S., Voragen A.G.J., Van Berkel W.J.H. (2006). Sorghum grain as human food in Africa: Relevance of content of starch and amylase activities—Review. Afr. J. Biotechnol..

[13] Taylor J.R.N., Belton P.S., Beta T., Duodu K.G. (2014). Increasing the utilization of sorghum, millets and pseudocereals: Developments in the science of their phenolic phytochemicals biofortification and protein functionality. J. Cereal Sci..

[14] Paiva C.L., Evangelista W.P., Queiroz V.A.V., Gloria M.B.A. (2015). Bioactive amines in sorghum: Method optimisation and influence of line, tannin and hydric stress. Food Chem..

[15] Kortei N.K., Odamtten G.T., Appiah V., Obodai M., Adu-Gyamfi A., Wiafe-Kwagyan M. (2015). Comparative occurrence of resident fungi on gamma irradiated and steam sterilized sorghum grains (*Sorghum bicolor* L.) for spawn production in Ghana. Br. Biotechnol..

[16] Abugri D.A., Akudago J.A., Pritchett G., Russell A.E., McElhenney W.H. (2015). Comparison of phytochemical compositions of *Sorghum bicolor* (L) Moench red flour and pale brown leaves. J. Food Sci. Nutr..

[17] Awika J.M., Rooney L.W., Wu X., Prior R.L., Cisneros-Zevallos L. (2003). Screening methods to measure antioxidant activity of sorghum (*Sorghum bicolor*) and sorghum products. J. Agric. Food Chem..

[18] Huang K.T., Weller C.L., Cuppett S.L., Hanna M.A. (2004). Policosanol contents and composition of grain sorghum kernels and dried distillers grains. Cereal Chem..

[19] Vita J.A. (2005). Polyphenols and cardiovascular disease: Effects on endothelial and platelet function. Am. J. Clin. Nutr..

[20] Kim E., Kim S., Park Y. (2015). Sorghum extract exerts cholesterol-lowering effects through the regulation of hepatic cholesterol metabolism in hypercholesterolemic mice. Int. J. Food Sci. Nutr..

[21] Kil H.Y., Seong E.S., Ghimire B.K., Chung I.M., Kwon S.S., Goh E.J., Heo K., Kim M.J., Lim J.D., Lee D.K. (2009). Antioxidant and antimicrobial activities of crude sorghum extract. Food Chem..

[22] Chen F., Cole P., Mi Z., Xing L.-Y. (1993). Corn and wheat-flour consumption and mortality from esophageal cancer in Shanxi, China. Int. J. Cancer.

[23] Isaacson C. (2005). The change of the staple diet of black South Africans from sorghum to maize (corn) is the cause of the epidemic of squamous carcinoma of the oesophagus. Med. Hypotheses.

[24] Van Rensburg S.J. (1981). Epidemiologic and dietary evidence for a specific nutritional predisposition to esophageal cancer. J. Natl. Cancer Inst..

[25] Yang L., Allred K.F., Geera B., Allred C.D., Awika J.M. (2012). Sorghum phenolics demonstrate estrogenic action and induce apoptosis in nonmalignant colonocytes. Nutr. Cancer.

[26] Costa L.M., Moura N.F., Marangoni C., Mendes C.E., Teixeira A.O. (2010). Atividade antioxidante de pimentas do gênero *Capsicum*. Ciência Tecnol. Aliment..

[27] De Orduna R.M. (2010). Climate change associated effects on grape and wine quality and production. Food Res. Int..

[28] Teixeira A., Eiras-Dias J., Castellarin S.D., Gerós H. (2013). Berry phenolics of grapevine under challenging environments. Int. J. Mol. Sci..

[29] Treutter D. (2001). Biosynthesis of phenolic compounds and its regulation in apple. Plant Growth Regul..

[30] Dong T.T., Cui X.M., Song Z.H., Zhao K.J., Ji Z.N., Lo C.K., Tsim K.W. (2003). Chemical assessment of roots of *Panax notoginseng* in China: Regional and seasonal variations in its active constituents. J. Agric. Food Chem..

[31] Choi S.C., Kim J.M., Lee Y.G., Kim C. (2019). Antioxidant activity and contents of total phenolic compounds and anthocyanins according to grain colour in several varieties of *Sorghum bicolor* (L.) Moench. Cereal Res. Commun..

[32] Davila-Gomez F.J. (2009). Evaluacion de la Produccion de Bioetanol a Partir del Kugo de Cinco Variedades de Sorgos Dulces y Forrajeros (*Sorghum bicolor* (L) Moench). Master’s Thesis.

[33] Luo X., Cui J., Zhang H., Yuqing Duan Y. (2018). Subcritical water extraction of polyphenolic compounds from sorghum (*Sorghum bicolor* L.) bran and their biological activities. Food Chem..

[34] Rhodes D.H., Stephen K. (2016). Sorghum [*Sorghum bicolor* (L.) Moench] genotypes with contrasting polyphenol compositions differentially modulate inflammatory cytokines in mouse macrophages. J. Chem..

[35] Nadeem M.A., Karakoy T., Yeken M.Z., Habyarimana E., Hatipoglu R., Çiftçi V., Nawaz M.A., Sonmez F., Shahid M.Q., Yang S.H. (2020). Phenotypic characterization of 183 Turkish common bean accessions for agronomic, trading, and consumer-preferred plant characteristics for breeding purposes. Agronomy.

[36] Martins S.R., Vences F.J., Miera L.E.S., Barroso M.R., Carnide V. (2006). RAPD analysis of genetic diversity among and within Portuguese landraces of common white bean (*Phaseolus vulgaris* L.). Sci. Hortic..

[37] Yanishlieva-Maslarova N.V., Heinonen I.M., Pokorny J., Yanishlieva N., Gordon M. (2001). Sources of natural antioxidants: Vegetables, fruits, herbs, spices and teas. Antioxidants in Food, Practical Applications.

[38] Sikora E., Cieslik E., Topolska K. (2008). The source of natural antioxidants. Acta Sci. Pol. Technol. Aliment..

[39] Mathew S., Abraham E.T. (2006). Studies on the antioxidant activities of cinnamon (*Cinnamonum verum*) bank extracts, through various in vitro models. Food Chem..

[40] Arts I.C.W., Hollman P.C.H. (2005). Polyphenols and disease risk in epidemiologic studies. Am. J. Clin. Nutr..

[41] Alfadda A.A., Sallam R.M. (2012). Reactive oxygen species in health and disease. J. Biomed. Biotechnol..

[42] Osawa T., Uritani I., Garcia V.V., Mendoza E.M. (1994). Novel natural antioxidants for utilization in food and biological systems. Postharvest Biochemistry of Plant Food-Materials in the Tropics.

[43] Shahidi F., Janitha P.K., Wanasundara P.D. (1992). Phenolic antioxidants. Crit. Rev. Food Sci. Nutr..

[44] Pietta P.G., Rice-Evans C.A., Packer L. (1998). Flavonoids in medicinal plants. Flavonoids in Health and Disease.

[45] Merken H.M., Beecher G.R. (2000). Measurement of food flavonoids by high performance liquid chromatography: A review. J. Agric. Food Chem..

[46] Fattouch S., Caboni P., Coroneo V., Tuberoso C.I.G., Angioni A., Dessi S., Marzouki N., Cabras P. (2007). Antimicrobial activity of tunisian quince (*Cydonia oblonga* Miller) pulp and peel polyphenolic extracts. J. Agric. Food Chem..

[47] Costa R.M., Magalhães A.S., Pereira J.A., Andrade P.B., Valentão P., Carvalho M., Silva B.M. (2009). Evaluation of free radical-scavenging and antihemolytic activities of quince (*Cydonia oblonga*) leaf: A comparative study with green tea (*Cammelia sinensis*). Food Chem..

[48] Velioglu Y.S., Mazza G., Gao L., Oomah B.D. (1998). Antioxidant activity and total phenolics in selected fruits, vegetables, and grain products. J. Agric. Food Chem..

[49] Parvaneh T., Abedi B., Davarynejad G.H., Moghadam E.G. (2019). Enzyme activity, phenolic and flavonoid compounds in leaves of Iranian red flesh apple cultivars grown on different rootstocks. Sci. Hortic..

[50] Scebba F., Sebustiani L., Vitagliano C. (1999). Protective enzymes against activated oxygen species in wheat (*Triticum aestivum* L.) seedlings: Responses to cold acclimation. J. Plant Physiol..

[51] Shigeoka S., Ishikawa T., Tamoi M., Miyagawa Y., Takeda T., Yabuta Y., Yoshimura K. (2002). Regulation and function of ascorbate peroxidase isoenzymes. J. Exp. Bot..

[52] Gołębiowska G., Wędzony M., Płażek A. (2011). The responses of pro and antioxidative systems to cold-hardening and pathogenesis differs in triticale (x *Triticosecale* Wittm) seedlings susceptible or resistant to pink snow mould (*Microdochium nivale* Fr., Samuels & Hallett). J. Phytopathol..

[53] Hanifei M., Dehghani H., Choukan R. (2013). The role of antioxidant enzymes and phenolic compounds in disease resistance to *Fusarium oxysporum* f. sp. *Melonis* race 1.2. Int. J. Agron. Plant Prod..

[54] Ivanov S., Miteva L., Alexieva V., Karjin H., Karanov E. (2004). Alterations in some oxidative parameters in susceptible and resistant wheat plants infected with *Puccinia recondita* f. sp. *tritici*. J. Plant Physiol..

[55] Kiraly L., Barna B., Kiraly Z. (2007). Plant resistance to pathogen infection: Forms and mechanisms of innate and acquired resistance. J. Phytopathol..

[56] Kumar M., Yadav V., Tuteja N., Johri A.K. (2009). Antioxidant enzyme activities in maize plants colonized with *Piriformospora indica*. Microbiology.

[57] Płażek A., Hura K., Żur I., Niemczyk E. (2003). Relationship between frost tolerance and cold-induced resistance of spring barley, meadow fescue and winter oilseed rape to fungal pathogens. J. Agron. Crop Sci..

[58] Shahidi F., Zhong Y. (2010). Novel antioxidants in food preservation and health promotion. Eur. J. Lipid Sci. Technol..

[59] Ghassemi-Golezani K., Asghar Aliloo A., Valizadeh M., Moghaddam M. (2008). Effects of different priming techniques on seed invigoration and seedling establishment of lentil (*Lens culinaris* Medik). J. Food Agric. Environ..

[60] Jung K.Y., Yun E.S., Park C.Y., Choi Y.D., Hwang J.B., Jeon S.H. (2012). Effects of seed size variation on germination and seeding vigour of sorghum (*Sorghum bicolor* L.). Korean J. Crop Sci..

[61] Harris D., Hamdi A., Terry A.C. (2006). Germination and emergence of Sorghum bicolor: Genotypic and environmentally induced variation in the response to temperature and depth of sowing. Plant Cell Environ..

[62] Mary S., Gopalan A. (2006). Dissection of genetic attributes yield traits of fodder cowpea in F3 and F4. J. Appl. Sci. Res..

[63] Alfieri M., Balconi C., Cabassi G., Habyarimana E., Redaelli R. (2017). Antioxidant activity in a set of sorghum landraces and breeding lines. Maydica.

[64] Elliott R., Mann L., Olfert L. (2007). Effects of seed size and seed weight on seedling establishment, seedling vigour and tolerance of summer turnip rape (*Brassica rapa*) to flea beetles, *Phyllotreta* spp.. Can. J. Plant Sci..

[65] Arystanbekkyzy M., Nadeem M.A., Aktas H., Yeken M.Z., Zencirci N., Nawaz M.A., Ali F., Haider M.S., Tunc K., Chung G. (2018). Phylogenetic and taxonomic relationship of turkish wild and cultivated emmer (*Triticum turgidum* ssp. dicoccoides) revealed by iPBSretrotransposons markers. Int. J. Agric. Biol..

[66] Ghasemi Pirbalouti A., Hashemi M., Taherian Ghahfarokhi F. (2013). Essential oil and chemical compositions of wild and cultivated *Thymus daenensis* Celak and *Thymus vulgaris* L.. Ind. Crops Prod..

[67] Mediani A., Abas F., Khatib A., Ping T.C., Lajis N.H. (2012). Influence of growth stage and season on the antioxidant constituents of *Cosmos caudatus*. Plant Food Hum. Nutr..

[68] Jugran A.K., Bahukhandi A., Dhyani P., Bhatt I.D., Rawal R.S., Nandi S.K. (2016). Impact of altitudes and habitats on valerenic acid, total phenolics, flavonoids, tannins, and antioxidant activity of *Valeriana jatamansi*. Appl. Biochem. Biotechnol..

[69] Dykes L., Rooney W.L., Rooney L.W. (2013). Evaluation of phenolics and antioxidant activity of black sorghum hybrids. J. Cereal Sci..

[70] Afify A.E.M.R., El Beltagi H.S., El Salam S.M.A., Omran A.A. (2012). Biochemical changes in phenols, flavonoids, tannins, vitamin E, carotene and antioxidant activity during soaking of three white sorghum varieties. Asian Pac. J. Trop. Biomed..

[71] Wu G., Johnson S.K., Bornman J.F., Bennett S.J., Fang Z. (2017). Changes in whole grain polyphenols and antioxidant activity of six sorghum genotypes under different irrigation treatments. Food Chem..

[72] Dia V.P., Pangloli P., Jones L., McClure A., Patel A. (2016). Phytochemical concentrations and biological activities of Sorghum bicolor alcoholic extracts. Food Funct..

[73] Barros F., Dykes L., Awika J.M., Rooney L.W. (2013). Accelerated solvent extraction of phenolic compounds from sorghum brans. J. Cereal Sci..

[74] Van-Der Sluis A., Dekker M., de Jager A., Jongen W. (2001). Activity and concentration of polyphenolic antioxidants in apple: Effect of cultivar, harvest year, and storage conditions. J. Agric. Food Chem..

[75] Iqbal S., Bhanger M.I. (2006). Effect of season and production location on antioxidant activity of *Moringa oleifera* leaves grown in Pakistan. J. Food Compos. Anal..

[76] Klochkova T.A., Kang S.H., Cho G.Y., Pueschel C.M., West J.A., Kim G.H. (2006). Biology of a terrestrial green alga *Chlorococcum* sp. (*Chlorococcales*, *Chlorophyta*) collected from the Miruksazi stupa in Korea. Phycologia.

[77] Zykova V.V., Grabel’nykh O.I., Turchaninova V.V., Antipina A.I., Koroleva N.A., Kolesnichenko A.V., Pobezhimova T.P., Konstantinov Y.M., Voinikov V.K. (2002). The effect of CSP310 on lipid peroxidation and respiratory activity in winter wheat mitochondria. Russ. J. Plant Physiol..

[78] Dubas E., Gołębiowska G., Żur I., Wędzony M. (2011). *Microdochium nivale* (Fr., Samuels & Hallett): Cytological analysis of the infection process in triticale (x Triticosecale Wittm.). Acta Physiol. Plant..

[79] Blokhina O., Virolainen E., Fagerstedt K.V. (2003). Antioxidants, oxidative damage and oxygen deprivation stress: A review. Ann. Bot..

[80] Chalker-Scott L. (1999). Environmental significance of anthocyanins in plant stress responses. Photochem. Photobiol..

[81] Jeon S.H., Kim I.S., Park S.K., Jung K.Y., Kim S.W., Son Y.S. (2017). Dependence of *Sorghum bicolor* antioxidant activity on harvest time. Sci. Asia.

[82] Manupriya B.R., Shenoy K.B., Patil S.L., Somashekarappa H.M. (2016). Effect of gamma radiation on total antioxidant capacity, total lipid concentration and shelf life of finger millet flour. J. Radiat. Cancer Res..

[83] Hussain P.R., Wani I.A., Suradkar P.P., Dar M.A. (2014). Gamma irradiation induced modification of bean polysaccharides: Impact on physicochemical, morphological and antioxidant properties. Carbohydr. Polym..

[84] Fombang E.N., Taylor J.R.N., Mbofung C.M.F., Minnaar A. (2005). Use of γ-irradiation to alleviate the poor protein digestibility of sorghum porridge. Food Chem..

[85] Przybylska-Balcerek A., Frankowski J., Szablewska K.S. (2020). The influence of weather conditions on bioactive compound content in sorghum grain. Eur. Food Res. Technol..

[86] Donno D., Cerutti A.K., Prgomet I., Mellano M.G., Beccaro G.L. (2015). Foodomics for mulberry fruit (*Morus* spp.): Analytical fingerprint as antioxidants’ and health properties’ determination tool. Food Res. Int..

[87] Dykes L., Rooney L.W., Waniska R.D., Rooney W.L. (2005). Phenolic compounds and antioxidant activity of sorghum grains of varying genotypes. J. Agric. Food Chem..

[88] Contreras J.J.L., García F.Z., Orona V.U., Ávila G.M., Rojas R., Medina G.N. (2015). Chromatic, phenolic and antioxidant properties of sorghum bicolor genotypes. Not. Bot. Horti Agrobot..

[89] Szymczycha-Madeja A., Welna M., Pohl P. (2012). Elemental analysis of teas and their infusions by spectrometric methods. Trends Anal. Chem..

[90] Valmorbida J., Boaro C.S.F., Scavroni J., David E.F.S. (2007). Crescimento de *Mentha piperita* L. cultivada em solução nutritiva com diferentes doses de potássio. Rev. Bras. Plantas Med..

[91] Kang J., Price W.E., Ashton J., Tapsell L.C., Johnson S. (2016). Identification and characterization of phenolic compounds in hydromethanolic extracts of sorghum wholegrains by LC-ESI-MS n. Food Chem..

[92] Zaroug M., Orhan I.E., Senol F.S., Yagi S. (2014). Comparative antioxidant activity appraisal of traditional Sudanese kisra prepared from two sorghum cultivars. Food Chem..

[93] Aoyama S., Yamamoto Y. (2007). Antioxidant activity and flavonoid content of welsh onion (*Allium fistulosum*) and the effect of thermal treatment. Food Sci. Technol. Res..

[94] Chung K.T., Wong T.Y., Wei C.I., Huang Y.W., Lin Y. (1998). Tannins and human health: A review. Crit. Rev. Food Sci. Nutr..

[95] John R.N., Anyango J.O. (2011). Sorghum Flour and Flour Products: Production, Nutritional Quality, and Fortification.

[96] Dykes L., Rooney L.W. (2006). Sorghum and millet phenols and antioxidants. J. Cereal Sci..

[97] De Morais Cardoso L., Pinheiro S.S., Martino HS D., Pinheiro-Sant’Ana H.M. (2017). Sorghum (*Sorghum bicolor* L.): Nutrients, bioactive compounds, and potential impact on human health. Crit. Rev. Food Sci. Nutr..

[98] Hahn D.H., Rooney L.W., Earp C.F. (1984). Tannins and phenols of sorghum. Cereal Foods World.

[99] Svensson L., Sekwati-Monang B., Lutz D.L., Schieber A., Ganzle M.G. (2010). Phenolic acids and flavonoids in nonfermented and fermented red sorghum (*Sorghum bicolor* (L.) Moench). J. Agric. Food Chem..

[100] Dicko M.H., Gruppen H., Traoré A.S., van Berkel W.J., Voragen A.G. (2005). Evaluation of the effect of germination on phenolic compounds and antioxidant activities in sorghum varieties. J. Agric. Food Chem..

[101] Singleton V.L., Rossi J.A. (1965). Colorimetry of Total Phenolics with Phosphomolybdic-Phosphotungstic Acid Reagents. Am. J. Enol. Vitic..

[102] Moreno MI N., Isla M.I., Sampietro A.R., Vattuone M.A. (2000). Comparison of the free radical-scavenging activity of propolis from several regions of Argentina. J. Ethnopharmacol..

[103] Xing Q., Kadota S., Tadata T., Namba T. (1996). Antioxidative effect of phenylethanoids from *Cistanche deserticola*. Biol. Pharm. Bull..

[104] Thaipong K., Boonprakob U., Crosby K., Cisneros-Zevallos L., Hawkins Byrne D. (2006). Comparison of ABTS, DPPH, FRAP, and ORAC assays for estimating antioxidant activity from guava fruit extracts. J. Food Compos. Anal..

